# miR-4716–3p and the target AKT2 Gene/rs2304186 SNP are associated with blood cancer pathogenesis in Pakistani population

**DOI:** 10.1016/j.ncrna.2024.03.005

**Published:** 2024-03-14

**Authors:** Jairus Olumasai Nandwa, Azhar Mehmood, Ishrat Mahjabeen, Kayode Yomi Raheem, Mamoudou Hamadou, Mouhamed Z.K.A. Raimi, Mahmood A. Kayani

**Affiliations:** a Department of Biosciences, COMSATS University Islamabad, Pakistan; b Department of Public Needs Research, Integrated Cancer Research Foundation of Kenya, Kenya; c Department of Genetics, Hebrew University of Jerusalem, Israel; d Department of Biological Sciences, Faculty of Science, University of Maroua, Cameroon

**Keywords:** AKT2, Blood cancer, Polymorphism, miRNA-4716–3p, Biomarkers

## Abstract

AKT2 is crucial for cancer cells' invasion, metastasis, and survival. It is a possible downstream gene target of cancer glycolysis-related microRNAs. The study investigated the role of miRNA-4716–3p, rs2304186, and the AKT2 gene in blood cancer pathogenesis. RT-qPCR was used to analyze AKT2 gene mRNA and miRNA-4716–3p expression in 200 blood cancer samples and 200 healthy controls. Furthermore, Tetra-ARMS PCR was used to examine the rs2304186 AKT2 SNP in 300 patients and 290 control samples. miRNA-4716–3p was shown to be significantly downregulated (p = 0.0294), whereas mRNA expression of the AKT2 gene was found to be significantly upregulated (p = 0.0034) in blood cancer patients compared to healthy individuals. miRNA-4716–3p downregulation (p = 0.0466) was more pronounced, while AKT2 upregulation was non-significant (p = 0.1661) in untreated patients compared to chemotherapy-treated patients. Blood cancer risk was significantly associated with the rs2304186 GT genotype (p = 0.0432), TT genotype (p = 0.0502), and mutant allele (T) frequency (p = 0.0008). Polymorphism rs2304186 was associated with an increased risk of blood cancer in dominant (p = 0.0011), recessive (p = 0.0502), and additive (p = 0.0008) genetic models. The results suggested that the rs2304186 and the deregulated expression of miRNA-4716–3p and AKT2 gene at the mRNA level may significantly increase the incidence of blood cancer, particularly in the Pakistani population. Therefore, these may function as suitable biomarkers for blood cancer diagnosis and prognosis. Additional, larger-scale investigations may be required to affirm these results.

## Graphical abstract

**Figure:**
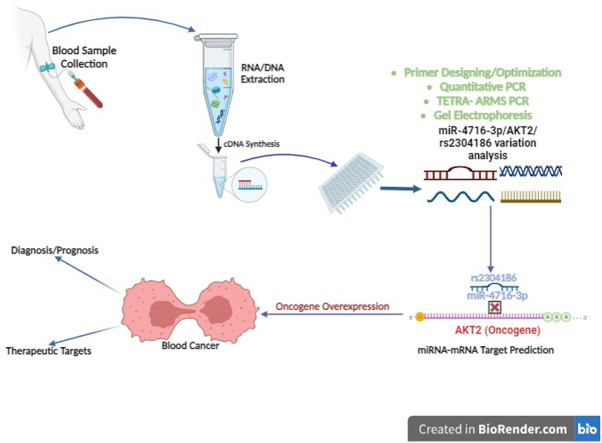

## List of abbreviations

ALL: Acute Lymphoblastic Lymphoma
AML: Acute Myeloid Leukemia
B-ALL: B-cell Acute Lymphoblastic Leukemia
CLL: Chronic Lymphocytic Leukemia
CML: Chronic Myeloid Leukemia
HL: Hodgkin Lymphoma
IDT: Integrated DNA Technology
MM: Multiple Myeloma
MREs: microRNA Recognition Elements
NHL: non-Hodgkin's Leukemia
NIHL: Noise-Induced Hearing Loss
NSCLC: Non-Small Cell Lung Cancer
SNPs: Single Nucleotide Polymorphisms
SPSS: Statistical Package for the Social Sciences
WHO: World Health Organization

## 1 Introduction

Blood cancer encompasses a variety of blood neoplasms frequently categorized by aberrant blood cell formation (hematopoiesis) [1]. World Health Organization (WHO) classification of hematological malignancies mainly depends on the cancerous cells' (myeloid or lymphoid) origin, whether those cells originated from the precursor, stem, differentiated, or committed cells, their growth rate, clinical, morphological, and genetic traits [2]. The most prevalent blood cancer types are leukemia, lymphoma, and myeloma, all beginning in the bone marrow and lymphoid system, respectively [3]. WHO reported 173,937 new cases of cancer in Pakistan in 2018, of which leukemia accounted for approximately 4.1%, non-lymphoma Hodgkin's (NHL) disease 3.4%, Hodgkin's lymphoma (HL) 0.92%, and multiple myeloma (MM) 0.81% [4]. In 2020, new leukemia cases in Pakistan increased by 4.7% and accounted for 5.7% of all cancer-related fatalities [5]. Environmental factors, including exposure to cancer-causing substances and lifestyle variables like smoking, are the main modifiable risk factors for blood cancer. The non-modifiable factors include rare genetic syndromes, age, gender, family history, and race [6,7].

miRNAs are noncoding RNAs that are short (18–23 nucleotides) and highly conserved. They attach to protein-coding transcripts' 3′-untranslated region (3′-UTR) to post-translationally control gene activity in multicellular organisms. miRNAs are expected to make up about 1–5% of human genomic areas, and these miRNAs modulate 30–60% of human genes that code for protein (Stark et al., 2010 [8]). miRNAs regulate essential biological functions such as differentiation, cell growth, and apoptosis. Tumor-suppressing microRNAs target oncoprotein-encoding mRNAs, while oncomiRNAs target antiapoptotic mRNAs. Multiple subtypes of blood cancer have been linked to abnormal microRNA expression. Transgenic mice overexpressing the human oncogene TCL1 demonstrate increased survival upon artificial stimulation of miR-181b expression, highlighting the role of miR-181b in the progression of (Chronic lymphocytic leukemia) CLL and its potential as a therapeutic target [9,10]. In HL cell lines and patients, tumor suppressors miR-203 and miR-34a undergo methylation. The re-expression of these microRNAs by demethylating agents results in an antiproliferative effect in HL cell lines [11]. In MDA-MB-231 cells, miR-7704 overexpression reduced cell division within the S/G2/M phases, thereby altering the malignant cell cycle. Moreover, the inhibition of miRNA-7704 resulted in reduced expression levels of HAGLR, indicating that miRNA-7704 targets HAGLR as a tumor suppressor [12].

Studies increasingly show that the tumor-aggressive oncogene AKT2 leads to cancer cell invasion, migration, and survival [13]. Overexpressing AKT2 in breast tumor cells increases their ability to metastasize in vivo by ensuring survival following invasion [14]. By explicitly targeting AKT2's 3′-UTRs, miR-497 inhibits AKT2 translocation, thus abating lung tumorigenesis and chemoresistance [15]. miR-218 epigenetic silencing influenced by *Rictor* upregulation initiates the mTOR-Akt signaling cascade, thereby contributing to oral carcinogenesis [16]. The 3′UTRs miRNA recognition elements (MREs) of genes often have single nucleotide polymorphisms (SNPs) that modify the target microRNA binding affinity resulting in gene expression dysregulation [17]. Ovarian cancer patients with the 3′ UTR rs4245739 SNP have a reduced chance of survival because it creates an unsuitable target site for miR-191. The rs16917496 T > C variant in SET8 is found within the miR502 3′UTR binding pocket which is associated with decreased inhibition in initial breast cancer [18]. Three miRNAs (miR-146a, 148, and 608) with SNPs rs2910164, rs11170877, and rs4919510, respectively, can influence the development of gastric cancer and its etiology [19]. miR-126 SNP detected in (Acute Lymphoblastic Leukemia) ALL patients block miRNA processing [20].

**Fig. 1:**
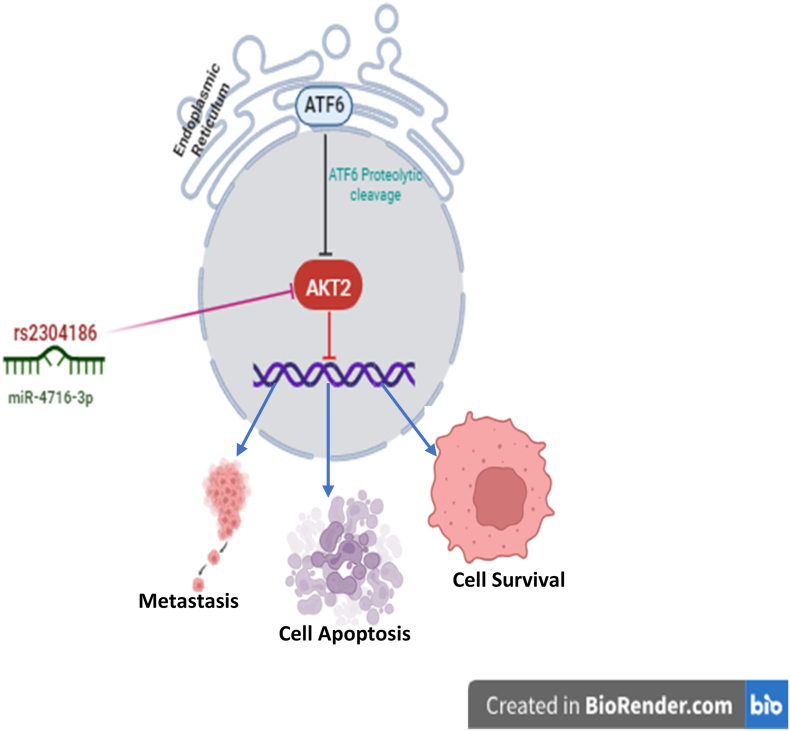
miR-4716–3p and AKT2 3' UTR SNP rs2304186 can promote cancer cell apoptosis, survival, and metastasis.

A single sequence location analysis determined that miRNA-4716–3p contains a purine-rich sequence with over 91% of investigated nucleotides [21]. miRNA-4716–3p differential expression emerges as a potential predictive biomarker for spinal chordoma patients [22], and colorectal cancer [23], and is noteworthy among the twenty miRNAs identified in Medaka (*Oryzias latipes*) ovarian tissues [24]. Yuanhuadine-resistant EGFR-TKI resistance was linked to miRNA-4716–3p downregulation in gef-resistant NSCLC cells [25]. The AKT2 gene 3′ UTR contains the SNP rs2304186, which, when mutated, prevents miRNA-4716–3p from binding (Fig. 1). According to a Chinese study in 2021, the AKT2 3′UTR SNP rs2304186 GT genotype increases noise-induced hearing loss (NIHL) risk. It caused an allele-specific change in the binding affinity of microRNA-625–5p to the modification site. Therefore, it may be used as a marker of susceptibility to NIHL [26]. In the Chinese Zhuang non-obesity population, rs2304186 increased the risk of polycystic ovary syndrome (PCOS) (J [27]). miRNA-4716–3p and AKT2 3′ UTR SNP rs2304186 (C→A) predicted mRNA interaction could be studied comprehensively across all cancer types [28]. No previous study has reported information on miRNA-4716–3p, AKT2 mRNA, and associated SNP (rs2304186) regulation in blood cancer patients in Pakistan. This study intended to examine the probability of miRNA-4716–3p targeting the AKT2 gene as a diagnostic and prognostic marker for blood cancer.

## 2 Materials and methods

### 2.1 Study subjects and ethical approval

Samples were collected from 150 to 300 blood cancers and healthy controls and matched backgrounds, ages, and sex. Table 1 lists the clinical and demographic details of blood cancer patients and controls. Blood cancer patients were histologically diagnosed between 2021 and 2022 at hospitals in Rawalpindi and Islamabad. Control samples were obtained from participants who were at these facilities for standard medical checkups. Gender, age, and residence-matched individuals were the inclusion criteria for the controls, whereas those with a history of cancer of any kind were excluded. Any additional conditions, such as diabetes, renal disease, or hepatic impairment were not included in the case and control groups. Informed consent was obtained from the participants. The on-the-spot filling of questionnaires from patients helped reduce biases. The designated hospitals and the Biosciences Department's Ethical Review Board at COMSATS University Islamabad authorized experimental protocols (Approval Number: CUI-Reg/Notif, 2928/22/3021). Every experiment was carried out in compliance with applicable or readily available rules and regulations (Declaration of Helsinki).

**Table 1:** Clinical and demographic characteristics.

Parameter	Cancer Patients	Controls	OR (95% CI)	p-value
**Age**				
Mean	33 ± 13	32 ± 13		
>33 n (%)	87 (43.5)	66 (33)	1.32 (1.02–1.70)	0.03
≤33 n (%)	113 (56.5)	134 (67)		
**Gender**				
Female n (%)	62 (31)	75 (37.5)	0.83 (0.63–1.09)	0.17
Male n (%)	138 (69)	125 (62.5)		
**Residence**				
Urban n (%)	94 (47)	83 (41.5)	1.13 (0.91–1.41)	0.27
Rural n (%)	106 (53)	117 (58.5)		
**Blood Cancer Types**				
Leukemia n (%)	170 (85)			
Lymphoma n (%)	20 (10)			
Myeloma n (%)	10 (5)			
**Types of Leukemia**				
ALL n (%)	84 (49.4)			
AML n (%)	43 (25.3)			
CLL n (%)	10 (5.9)			
CML n (%)	33 (19.4)			
**Lymphoma Types**				
HL n (%)	11 (55)			
NHL n (%)	9 (45)			
**Addiction**				
Smokers n (%)	132 (66)			
Non-Smokers n (%)	68 (34)			
**Familial History**				
Familial n (%)	54 (27)			
Non-Familial n (%)	146 (73)			
**Treatment**				
Chemotherapy n (%)	169 (84.5)			
Chemo + Radiotherapy n (%)	7 (3.5)			
Untreated n (%)	24 (12)			

n, number; ALL. Acute lymphoblastic leukemia; AML, Acute Myeloid Leukemia; CLL, Chronic lymphoblastic leukemia, CML, Chronic myeloid leukemia.

### 2.2 Sample collection and processing

Patients and controls underwent standard venipuncture to collect 5 mL of peripheral blood in EDTA-coated tubes, which were then kept at −20 °C awaiting processing.

### 2.3 Expression analysis of miRNA-4716–3p and *AKT2* gene

#### 2.3.1 RNA extraction

TRIzol (Guanidinium thiocyanate-phenol-chloroform) reagent (Invitrogen, CA, United States) was utilized to extract RNA [29]. In a brief stepwise procedure, after adding 500–700 l of Trizol®, the blood samples were gently mixed to achieve homogeneity. The samples were transferred to an Eppendorf tube (1.5 ml), nucleoprotein complexes, and allowed to dissolve fully through a 5-min room-temperature incubation. Following incubation, 200 μl of chloroform was added to the test tube. The tubes underwent an inversion shake for about 30 secs before undergoing a 5-min room-temperature incubation. The mixture was centrifuged at 4 °C for 15 min at 12,000 rpm. The solution was separated into three distinct phases containing RNA, DNA, and proteins, respectively: the upper aqueous phase, the middle phase, and the lower phase. The upper transparent RNA layer was extracted and transferred to a separate Eppendorf tube carefully. Following a room temperature incubation of 15 min, the samples were spun for 10 min at 13,000 rpm and 4 °C after adding 500 μl chilled isopropanol. After centrifugation, an RNA precipitate/pellet was formed at the bottom of the tube. The resultant supernatant was discarded, and the RNA particle was washed with 1 mL of 70% alcohol. The tubes were centrifuged at 4 °C for 10 min at 10,000 rpm. After supernatant removal, the particle was allowed to air dry for 15 min. Finally, the RNA residue was dissolved in Diethylpyrocarbonate (DEPC) treated water. Both short-term (at −20 °C) and long-term (at −80 °C) storage conditions were used for the isolated RNA. A Nanodrop spectrophotometer was used to check the RNA's concentration.

#### 2.3.2 cDNA synthesis

cDNA was synthesized from the extracted RNA using the High-capacity cDNA reverse transcription kit. The cDNA synthesis was performed in a single step [30]. Regardless of the extracted RNA concentration, each sample's total volume was less than 20 μl. A 0.2 ml centrifuge tube was filled with 1 μl of RNA, 10 μl of nuclease-free water, 2 μl of dNTPs, 5 μl of 5x reaction buffer, 1 μl of Random Hexamer Primer, 1 μl of RNA inhibitor and 1 μl of Reverse Aid RT. The tubes were incubated at 25 °C for 5 min, followed by 42 °C for 60 min, and then 80 °C for 5 min. The reaction's products were chilled on ice and briefly centrifuged. The products were used immediately for PCR or stored at −20 °C until further processing.

#### 2.3.3 Primer designing

The microRNA database (miRBase) was queried for the microRNA-4716–3p information. The Integrated DNA Technology (IDT) software's primer quest tool was used to generate primers, which were then validated using UCSC In-silico PCR and NCB1 PrimerBlast [31]. For the design of β-actin, U6, and AKT2 primers, the coding sequence was downloaded from the Ensemble Genome Browser, with the remaining steps following the above microRNA primer designing pattern. The selected primers are reported in Table S1.

#### 2.3.4 Quantitative real-time PCR (polymerase chain reaction)

The microRNA microRNA-4716–3p and its target gene (AKT2) expression were analyzed using the qPCR (quantitative PCR). Internal standards, U6 and β-actin, were also employed. This was accomplished by following the ABI PRISM 7000 Sequence Detection System's supplier's directives (Applied Biosystems) [32]. Each tube contained 1 μl of cDNA, 4 μl of RNAse-free water, 4 μl 2X SYBR Green master mix, 1 μl forward Primer, and 1 μl reverse primer. The qPCR cycling conditions were 10 min at 95 °C, 35 cycles for 15 secs at 95 °C, and a 60°C-annealing temperature for 1 min. The relative expression levels of microRNA-4716–3p and the AKT2 gene were evaluated using the 2−ΔΔCt method.

### 2.4 AKT2 gene SNP rs2304186 methodology

#### 2.4.1 DNA extraction

Genomic DNA was extracted using the phenol-chloroform method [33]. A nanodrop spectrophotometer (ND-1000, USA) and 1% gel electrophoresis were used to quantify the isolated DNA [29,34]. Electropherograms of the patient and control DNA samples are displayed in Fig. S11.

#### 2.4.2 Polymorphism selection and primer designing

The dsSNP NCBI and Ensembl Genome Browser databases accessible at (https://www.ncbi.nlm.nih.gov/snp/) and (https://asia.ensembl.org/index.html), respectively, were queried to obtain the rs2304186 SNP. Conditions for polymorphism inclusion included; Minutes or allelic frequency of greater than 5% (two) SNP in distinct gene areas to account for the variation impacts and were excluded from the existing CRC literature (M.-Y [35]). UCSC in-silico PCR (https://genome.ucsc.edu/cgi-bin/hgPcr) and NCBI Primer-BLAST (https://www.ncbi.nlm.nih.gov/tools/primer-blast/) confirmed tetra primer sets for specific SNPs generated by Primer 1 software (http://primer1.soton.ac.uk/primer1.html) [33]. Table S2 shows SNP 2304186 primer sequences.

#### 2.4.3 Tetra-ARMS PCR (polymerase chain reaction)

The Refractory Mutation System (ARMS) [33] was used to amplify the rs2304186 SNP. The PCR tube contained 1 μl DNA template, 1.5 μl reverse outer/inner and 1.5 μl forward outer/inner primers, 3 μl PCR water, and 6 μl PCR master mix. The conditions for PCR were set as initial denaturation (5 min at 95 °C), 35 cycles of denaturation (45 s at 95 °C), and elongation (1 min at 59 °C). A 2% gel electrophoresis was used to visualize the PCR-generated product (J [27]). Fig. S12 displays the electropherograms of PCR-amplified products of patients and control blood samples.

### 2.5 Statistical analysis

Age, gender, and histology data of patients and healthy individuals, and the comparative fold change in gene and miRNA expression were distinguished using an ANOVA and a one-sample *t*-test. The diagnostic utility of miR-4716–3p and the AKT2 gene was evaluated using a receiver operating characteristic (ROC) curve analysis. An analysis of the variations in genotypes and allele frequencies between case group studies and control samples was conducted using a two-way ANOVA and the χ2 test. A logistic regression (LR) model was employed to estimate odds ratios (ORs) with corresponding 95% confidence intervals (CIs) and to correlate the SNP with blood cancer demographic and clinical parameters. A P value < 0.05 was considered statistically significant among all groups. Graph Pad Prism software, Microsoft Excel, version 21.0 of the Statistical Package for the Social Sciences (IBM SPSS Statistics), and MedCalc Software were used to generate tabular and graphical presentations.

## 3 Results

### 3.1 Clinical and demographic data of cancer patients

The current study included 200 blood cancer-diagnosed patients and a similar number of healthy individuals. RT-qPCR technique was used to analyze AKT2 gene mRNA expression and microRNA-4716–3p expression. Table 1 provides in-depth demographic and clinical information on the study cohort.

### 3.2 AKT2 gene association with blood cancer

**Fig. 2:**
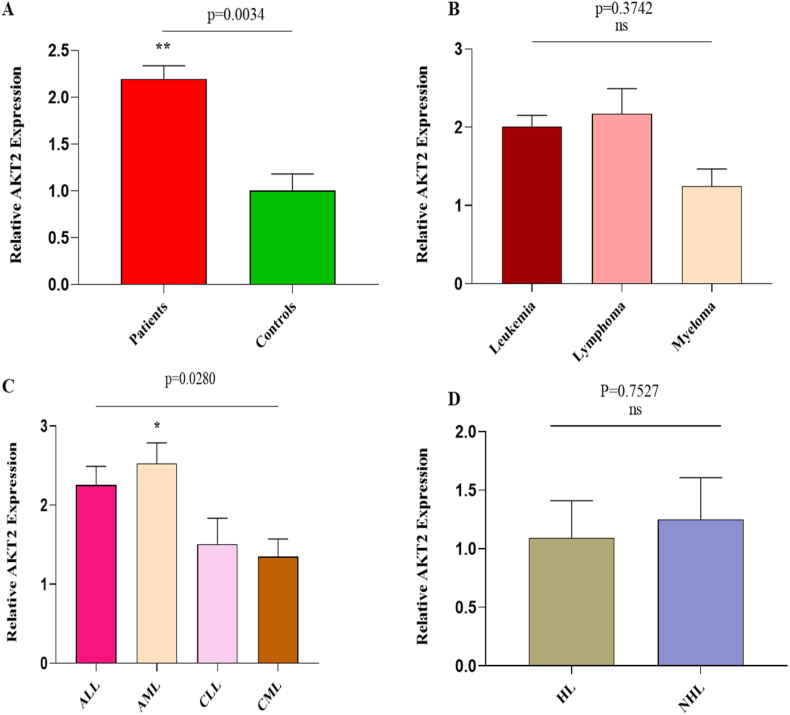
**AKT2 Association with Blood Cancer** (A) AKT2 gene expression in blood cancer patients versus healthy controls (B) Relative expression of AKT2 gene in different types of blood cancer patients. (C) Relative expression of AKT2 gene in leukemia sub-types (D) Relative expression of AKT2 gene in lymphoma types.

The student t-test was applied for the statistical evaluation showed the relative AKT2 gene mRNA expression was significantly upregulated (p = 0.0034) in the patient's cohort compared to healthy individuals (Fig. 2A). Patients in the study population were characterized into diverse groups based on types of blood cancer, including leukemia, myeloma, and lymphoma. ANOVA-based analysis showed a non-significant downregulation of AKT2 gene expression in myeloma compared to leukemia and lymphoma (p = 0.3742) (Fig. 2B). Different types of leukemia were also catalogued in the data gathered from patients with blood cancers, and the levels of AKT2 gene expression between different categories of leukemia were assessed. The results revealed a significant upregulation of the AKT2 gene in AML patients (p = 0.0280) compared to ALL, CLL, and CML (Fig. 2C). Patients reported having HL, as well as NHL lymphomas. The student t-test results revealed a non-significant upregulation of AKT2 gene expression in NHL patients (p = 0.7527) compared to HL patients (Fig. 2D).

### 3.3 Association of miRNA-4716–3p with blood cancer

**Fig. 3:**
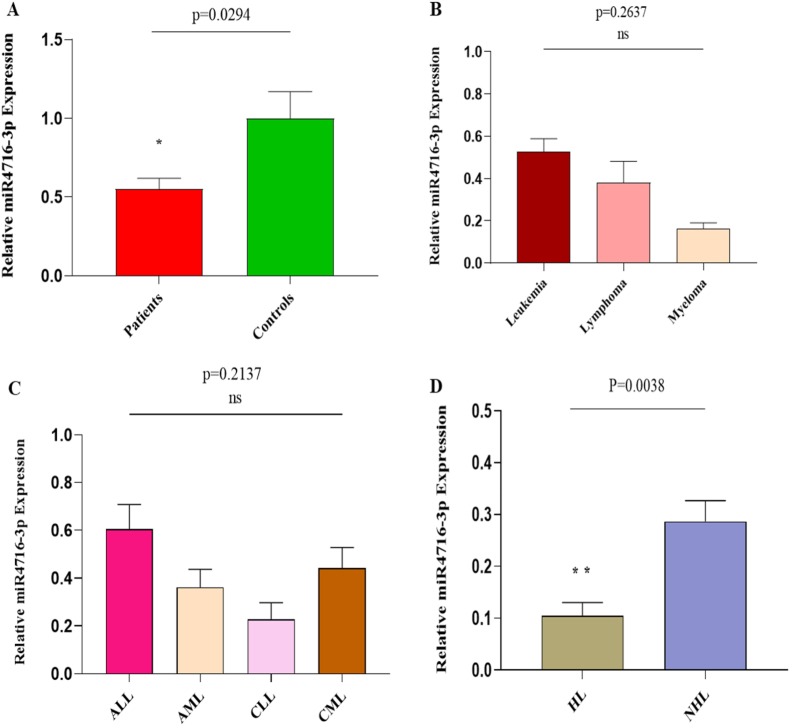
**Association of miRNA-4716-3p with Blood Cancer** (A) Relative expression of microRNA- 4716–3p in Blood Cancer Patients and Controls (B) microRNA-4716–3p expression levels in different types of blood cancer (C) Relative expression of microRNA- 4716–3p in leukemia sub-types (D) Expression of microRNA-4716–3p in lymphoma types.

MicroRNA-4716–3p expression levels were significantly downregulated (p = 0.0294) in patients compared to healthy controls (Fig. 3A). ANOVA analysis showed that microRNA-4716–3p expression was non-significantly downregulated (p = 0.2637) in myeloma compared to leukemia and lymphoma (Fig. 3B). In contrast to ALL, AML, and CML, microRNA-4716–3p expression showed a non-significant downregulation in CLL patients (p = 0.2137) (Fig. 3C). However, in lymphomas, there was significant downregulation (p = 0.0038) of microRNA-4716–3p expression in HL patients compared to NHL patients (Fig. 3D).

### 3.4 miRNA-4716–3p and AKT2 gene diagnostic value

**Fig. 4:**
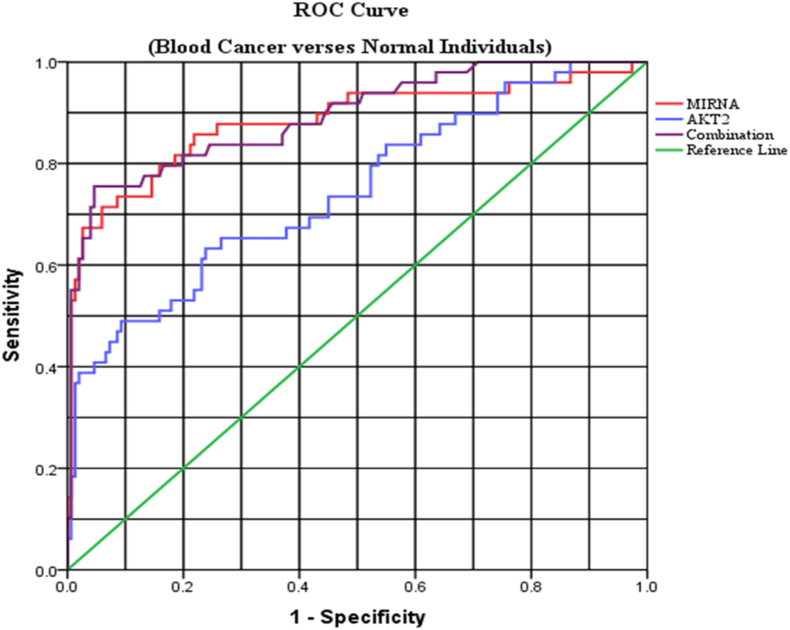
miR-4716–3p and AKT2 gene Diagnostic Value Analysis.

ROC curve analysis was used to evaluate the diagnostic power of miRNA-4716–3p and its target AKT2 gene (Fig. 4). Using the area under the curve (AUC) for diagnostic assessment, the cutoff values were 0.208 and 0.0369 for miRNA-4716–3p and the AKT2 gene, respectively. The AUCs for miRNA-4716–3p and AKT2 gene were 0.882 (p < 0.0001), and 0.745 (p < 0.0001), respectively (Table 2). With an ideal sensitivity and specificity of 100% and 99%, respectively, the combination of the AKT2 gene and miRNA-4716–3p demonstrated exceptional discriminatory ability in differentiating blood cancer from healthy individuals, with an AUC of 0.893 (p < 0.0001).

**Table 2:** miRNA-4716–3p and AKT2 Gene ROC Details.

miRNA/Gene	Sensitivity	Specificity	AUC	p-value	Cutoff	95% CI
miR-4716–3p	0.96	0.76	0.882	<0.0001	0.208	0.816–0.948
AKT2 Gene	0.98	0.86	0.745	<0.0001	0.369	0.661–0.828
Combination	1	0.99	0.893	<0.0001	2.01	0.836–0.949

AUC, Area Under the curve; C1, Confidence Interval; p-value ≤0.05 considered statistically significant.

### 3.5 Correlation between AKT2 gene and miRNA-4716–3p and blood cancer demographic and clinical characteristics

The relative expression of the AKT2 gene was correlated with various histopathological parameters as shown in Table 3. ANOVA and student t-test analysis were performed and the results showed a significant correlation between the AKT2 gene and rural-based patients (p = 0.0310) (Fig. S1C), and patients with no blood cancer family history (p = 0.0270) (Fig. S1F). A similar positive correlation was detected between miRNA-4716–3p and rural-based (p = 0.0428), female (p = 0.0423), smoking (p = 0.0425), treated (p = 0.0466), and employed (p = 0.0460) patients (Fig. S2). Table 3 shows that miRNA-4716–3p had a non-significant association with age, familial history, and marital status, while the AKT2 gene showed non-significant expression relating to age, gender, addiction, treatment, and occupation.

**Table 3:** Correlation Between AKT2 Gene and miR-4716–3p and Blood Cancer Demographic and Clinical Characteristics.

AKT2 Gene			
PARAMETERS	S.E	95% CI	p-value
AGE	0.53	−1.37–0.71	0.5399 (ns)
GENDER	0.61	−1.28–1.12	0.8922 (ns)
RESIDENCE	0.46	0.09–1.89	0.0310 (s)
ADDICTION	0.53	−1.41–0.69	0.5073 (ns)
TREATMENT	0.24	−2.21–0.39	0.1661 (ns)
FAMILIAL HISTORY	0.56	0.15–2.35	0.0270 (s)
OCCUPATION	0.54	−1.10–1.03	0.9468 (ns)
**MIR-4716-3P**			
AGE	0.14	−0.20–0.36	0.5891 (ns)
GENDER	0.02	−1.04–0.38	0.0423 (s)
RESIDENCE	0.19	−0.74–0.83	0.0428 (s)
ADDICTION	0.01	−1.08–0.27	0.0425 (s)
TREATMENT	0.10	−0.64–0.79	0.0466 (s)
FAMILIAL HISTORY	0.18	−0.07–0.64	0.1151 (ns)
OCCUPATION	0.02	−0.03–0.38	0.0460 (s)

ns, non-significant; s, significant; SE, Standard Error; CI, confidence interval; p-value ≤0.05 considered statistically significant; n, number of samples.

### 3.6 Association of AKT2 gene polymorphism (rs2304186) with blood cancer

The observed genotype and allele frequencies in patients and control samples were used in the rs2304186 association study. The analyses showed that heterozygous genotype (GT) (OR: 1.3992; 95% CI (1.0104–1.9375); p = 0.0432) and homozygous mutant genotype (TT) (OR: 1.7051; 95% CI (0.9995–2.909; p = 0.0502) were significantly linked with increased risk of blood cancer when compared to controls. Moreover, in blood cancer samples, the frequency of the mutant allele (T) (OR: 1.5157; 95% CI (1.1877–1.9344); p = 0.0008) of SNP rs2304186 was significantly higher than in healthy controls (Table 4).

**Table 4:** Genotypic and allelic frequencies of AKT2 gene SNP (rs2304186) in blood cancer.

Genotype/Allele	Patients n (%)	Control n (%)	OR (95% CI)	P-value
GG (W)	112 (37)	147 (51)	1	1
GT (H)	148 (49)	119 (41)	1.3992 (1.0104–1.9375)	0.0432
TT (M)	40 (14)	24 (8)	1.7051 (0.9995–2.909)	0.0502
G	372 (62)	413 (71)	1	1
T	228 (38)	167 (29)	1.5157 (1.1877–1.9344)	0.0008

CI, confidence interval; OR, odds ratio; p-value ≤0.05 considered statistically significant; n, number of samples.

### 3.7 The association between rs2304186 and blood cancer risk in three genetic models

Genetic models are computed in genetically related studies for determining vulnerability to disease loci utilizing statistical power. Patient and control populations' genetic frequencies were further evaluated using dominant, recessive, and additive genetic association models. Polymorphism rs2304186 exhibited significant correlations with higher blood cancer risk in dominant (OR: 1.7255; 95% CI (1.2427–2.3959); p = 0.0011), recessive (OR: 1.7051; 95% CI (0.9995–2.909; p = 0.0502), and additive (OR: 1.5157; 95% CI (1.1877–1.9344); p = 0.0008) models (Table 5).

**Table 5:** Genetic model calculations of rs2304186 SNP in blood cancer patients.

Models	Cases n (%)	Control n (%)	OR (95% CI)	P-value
Dominant (GG vs GT + TT)	188 (63)	143 (49)	1.7255 (1.2427–2.3959)	0.0011
Recessive (TT vs GG + GT)	40 (14)	24 (8)	1.7051 (0.9995–2.909)	0.0502
Additive (T vs G)	228 (38)	167 (29)	1.5157 (1.1877–1.9344)	0.0008

CI, confidence interval; OR, odds ratio; p-value ≤0.05 considered statistically significant; n, number of samples.

### 3.8 Correlation of AKT2 gene SNP (rs2304186) with histopathological parameters of blood cancer patients

The link between the genotype AKT2 SNP rs2304186 and blood cancer histopathological characteristics is shown in Table S3. A non-significant risk effect of homozygous mutant genotype (TT) (OR; 1.1317; 95% CI (0.5798–2.2088); P = 0.7169) was more evident in patients in the ≤33 age group compared to > 33 age group patients. Heterozygous genotype (GT) (p = 0.2078) and mutant allele (T) (p = 0.5761), showed a non-significant decreased risk of blood cancer in the ≤33 age group. The homozygous mutant genotype was associated with a non-significant elevated risk among females (OR; 1.1058; 95% CI (0.5676–2.1545); p = 0.7675), and a reduced risk in addicted blood cancer patients (OR; 0.5224; 95% CI (0.2636–1.0352); p = 0.0627). In comparison to non-smokers, the heterozygous genotype (GT) has a non-significant higher risk of blood cancer in smokers (OR: 1.2510; 95% CI (0.7763–2.0159); p = 0.3577). The heterozygous genotype (GT) was non-statistically associated with a lower frequency and risk in females compared to males (OR: 0.8466; 95% CI (0.537–1.3345); p = 0.4732). Addicted patients (OR = 0.8055, 95% CI = 0.5707 to 1.137, p = 0.2187) and females (OR = 0.9619, 95% CI = 0.6906 to 1.3397, p = 0.8183) showed a non-significantly decreased risk of developing blood cancer due to the mutant allele (T). The results showed a non-significant association and protective role for the heterozygous (GT) genotype and with the presence of leukemia (p = 0.0645), lymphoma (p = 0.2732), or myeloma (p = 0.3766) patients. The homozygous mutant genotype was associated with a significant increase in leukemia risk (OR: 1.8379; 95% CI (1.053–3.2079); p = 0.0322) but a non-significant increase in lymphoma (p = 0.7502) and decrease in myeloma (p = 0.9174) risk. The mutant allele (T) showed a significant increase in blood cancer risk in leukemia patients compared to controls (OR: 1.5674; 95% CI: 1.2096 to 2.0312); p = 0.0007). The heterozygous genotype was negatively associated with patients' marital status (p = 0.1264), location (p = 0.8605), therapy (p = 0.1834), and family history of blood cancer (p = 0.0627). Homozygous mutant genotype showed a non-significant association with increased risk of blood cancer with patients' residence (OR; 1.1773; 95% CI (0.5957–2.3269); p = 0.6387) and treatment (OR; 1.1718; 95% CI (0.5303–2.5896); p = 0.6951), and decreased risk of blood cancer with patients' marital status (OR; 0.7674; 95% CI (0.3877–1.519); p = 0.4473) and familial history (OR; 0.8353; 95% CI (0.4225–1.6514); p = 0.6048). A non-significant correlation was observed between the mutant allele (T) and blood cancer patients’ residence (rural/urban) (p = 0.5530), treatment (p = 0.5602), familial history (p = 0.3975), and marital status (p = 0.7185).

## 4 Discussion

Blood cancers present a formidable challenge due to their unpredictable natural history and prognosis, underscoring the critical need for identifying novel biomarkers to enhance early detection and therapy response prediction. In this study, we specifically investigated the microRNA-4716–3p, its target AKT2 gene, and rs2304186 variations of blood cancer patients alongside healthy individuals to discern potential biomarkers for non-invasive prognostic and diagnostic purposes.

Demographic analysis (Table 1) revealed a higher incidence of blood cancer in males (69%) compared to females (31%), echoing trends observed in Pakistan research where males constituted 55.7% of blood cancer patients while females constituted 44.3% [36]. Rural areas exhibited a higher prevalence of blood cancer cases (53%) compared to urban locales (47%). Blood cancer diagnoses span all age groups, with incidence typically rising with age. Our cohort had a higher incidence observed in individuals ≤33 years (56.5%) compared to those >33 years (43.5%). Similar age distributions were noted in Pakistani studies [37], and in Indian AML patients [38], with presentation ages around 32- and 38-years. Leukemia emerged as the most prevalent blood malignancy (85%), followed by lymphoma (10%) and myeloma (5%). ALL comprised 49.4% of all leukemia diagnoses, while AML constituted 25.3%, CML 19.4%, and CLL 5.9%. Arshad et al. [39] reported AML to account for 50% of all leukemia types among the Pakistani population. 73% of blood cancer patients had no previous familial predisposition, whereas 23% exhibited familial antecedents, aligning closely with [40] findings that documented 25.6% of cancer participants having a family history.

The latest research has shown AKT2 as an oncogenic gene that fosters cancer cell longevity and invasion, contributing to tumor aggressiveness. Higher AKT2 expression levels are associated with lung cancer [41], and osteosarcoma [42]. Our results indicated a significant upregulation of AKT2 mRNA levels in blood cancer patients compared to healthy controls. Specifically, AML showed higher expression levels of AKT2 compared to other leukemia types, suggesting its potential role in blood cancer pathogenesis. Conversely, myeloma exhibited a non-significant downregulation of AKT2 compared to leukemia and lymphoma, as well as in HL compared to NHL. Previous findings reported that rectal cancer survival [23], and prostate cancer development [43] correlated with downregulated microRNA-4716–3p expression levels. microRNA-4716–3p in our study demonstrated significant downregulation in blood cancer patients, particularly untreated individuals, compared to those who received chemotherapy or radiotherapy. Whereas leukemia types exhibited non-significant microRNA-4716–3p expression levels, HL showed lower expression compared to NHL. Furthermore, while both the AKT2 gene and miRNA-4716-3p are capable of differentiating between blood cancer patients and healthy individuals on their own, our findings indicated that the two combined exhibited improved diagnostic efficacy (Fig. 4). Notably, AKT2 and microRNA-4716–3p expression levels varied across demographic and clinical factors including age, gender, residence, addiction, familial history, treatment, and smoking status (Table 2; Fig. S1; Fig. S2), suggesting their promise as a marker for assessing blood cancer risk.

Several human disorders, including gastric cancer [44], NIHL [26], and PCOS [45] have been linked to rs2304186 AKT2 gene polymorphisms. We observed a higher risk of blood cancer development associated with the rs2304186 GT/TT variants and the mutant allele (T). Genetic models conferred an elevated risk of blood cancer development, particularly in the dominant, recessive, and allelic models (Table 4). The rs2304186 GT/TT genotypes and T allele had non-significant associations with increased blood cancer risk across age and gender subgroups. Individuals over 35 years with rs2304186 GT/TT were reported to have higher NIHL risks [26], indicating age's role in susceptibility to sickness, including blood cancer. Addiction, marital status, residence, and family history weren't significantly linked to the AKT2 rs2304186 GT genotype and blood cancer. However, the GT genotype protected against blood cancer recurrence post-treatment. The TT genotype didn't offer significant protection against addiction, marital status, or family history, but increased blood cancer risk with residence and therapy by 1.1773 and 1,1718 folds, respectively. The mutant (T) allele was associated with marginally higher blood cancer risk with marital status, location, and family history and lower risk with treatment, addiction, gender, and age. Blood cancer correlation studies support the results of the gene-by-environment interactions [41,46,47].

The rs2304186 GT genotype had a non-significant increased risk for leukemia (1.3883-fold), and lymphoma and myeloma (1.4370-fold). The TT genotype showed a non-significant higher risk for leukemia (1.84-fold) and lymphoma (1.19-fold). The T allele significantly increased leukemia risk by 1.56-fold (p = 0.0007), with slight increases for lymphoma and myeloma by 1.45 and 1.21-fold, respectively. Our study underscores the potential utility of AKT2 and microRNA-4716–3p as biomarkers for blood cancer prognosis and diagnosis. Additionally, the association between the AKT2 gene polymorphism and blood cancer susceptibility highlights the role of genetic factors in disease pathogenesis.

## 5 Conclusion

The 3′UTR is largely believed to have a pivotal function in controlling mRNA stability, translation, and subcellular localization. Previous research has shown that SNPs in the 3′UTR miRNA-binding domain can disrupt miRNA's biological function by creating or eliminating the target gene mRNA's miRNA binding site, causing cellular malfunction and illness, including cancer. Our findings propose that microRNA-4716–3p may contribute to the development of blood cancer by targeting and controlling the expression of the AKT2 gene, a process that may be modulated by the rs2304186 polymorphism. However, these results depended on other risk variables like age, gender, and geographic location.

While the assessments were extensive, they do have some caveats. Blood cancer is a complicated disease to be attributed to a single gene, microRNA, or SNP. Therefore, more loci and genes need to be taken into account. In vivo and in vitro research may be required to confirm our observational association studies and reveal the molecular mechanism of the AKT2 rs2304186 SNP's effects on blood cancer risk.

## Funding

O.N. Jairus was supported by Queen Elizabeth Commonwealth scholarship for his master's studies in Pakistan. He received a master's research grant from the 10.13039/501100000531Association of Commonwealth Universities, UK to COMSATS University Islamabad (Ref No: 2022-293-141-007664).

## CRediT authorship contribution statement

**Jairus Olumasai Nandwa:** Writing – review & editing, Writing – original draft, Investigation, Formal analysis, Conceptualization. **Azhar Mehmood:** Writing – original draft, Formal analysis, Conceptualization. **Ishrat Mahjabeen:** Resources, Formal analysis, Data curation. **Kayode Yomi Raheem:** Software, Formal analysis. **Mamoudou Hamadou:** Writing – review & editing, Writing – original draft, Formal analysis. **Mouhamed Z.K.A. Raimi:** Writing – original draft, Resources. **Mahmood A. Kayani:** Validation, Supervision.

## Declaration of competing interest

The authors declare no conflict of Interest. Thus, they approved the submission and publication of this manuscript without any reservations.
