# Supplementary material for: miR-4716–3p and the target AKT2 Gene/rs2304186 SNP are associated with blood cancer pathogenesis in Pakistani population
Source: Noncoding RNA Res. 2024 Mar 14;9(3):695–703. doi: 10.1016/j.ncrna.2024.03.005 (PMC10990746; doi:10.1016/j.ncrna.2024.03.005)
Supplement: Multimedia component 1 [file mmc1.docx]

**Supplementary data for**

**miR-4716-3p/rs2304186 SNP and the Target AKT2 Gene is Associated with Blood Cancer Pathogenesis in Pakistani Population**

Nandwa Olumasai Jairus1,2,3⃰, Azhar Mehmood1, Ishrat Mahjabeen1, Kayode Yomi Raheem1, Hamadou Mamoudou4, Mouhamed Z K A Raimi1, Mahmood A. Kayani1.

1 Department of Biosciences, COMSATS University Islamabad, Pakistan.

2 Department of Public Needs Research, Integrated Cancer Research Foundation of Kenya.

3 Department of Genetics, Hebrew University of Jerusalem, Israel.

4 Department of Biological Sciences, Faculty of Science, University of Maroua, Cameroon.

* Corresponding Author email: [jairus.nandwa@cancerresearchkenya.org](mailto:jairus.nandwa@cancerresearchkenya.org)

**Table S1:** Primer sequences of β-actin, U6, microRNA-4716-3p, and AKT2

| **Gene** | **Primer** | **Sequence** | **Annealing**  **Temperature** | **Product**  **Length** |
| --- | --- | --- | --- | --- |
| β-actin | Forward | 5’- TTCTCTGACCTGAGTCTCCTT-3’ | 55°C | 116bp |
|  |  |  |  |  |
|  |  |  |  |  |
|  | Reverse | 5’- ACACCCACAACACTGTCTTAG-3’ | 55°C | 116bp |
|  |  |  |  |  |
|  |  |  |  |  |
| AKT2 | Forward | 5’-TGTCTCCATGTTTCCTTCCC-3’ | 61°C | 77bp |
|  |  |  |  |  |
|  | Reverse | 5’-CACACTTTGTCTCCATGTTTCC-3’ | 61°C | 77bp |
|  |  |  |  |  |
|  |  |  |  |  |
| U6 | Forward | 5’-GCTTCGGCAGCACATATACTA-3’ | 62°C | 88bp |
|  |  |  |  |  |
|  |  |  |  |  |
|  | Reverse | 5’- CGAAGCCGTCGTGTATATGAT-3’ | 62°C | 88bp |
|  |  |  |  |  |
|  |  |  |  |  |
| miRN | Forward | 5’-TGTCTCCATGTTTCCTTCCC-3’ | 61°C | 77bp |
| A- |  |  |  |  |
| 4716- | Reverse | 5’- CACACTTTGTCTCCATGTTTCC-3’ | 61°C | 77bp |
| 3p |  |  |  |  |
|  |  |  |  |  |

**Table S2:** rs2304186 Primer Sequences

| **SNP** | **Primer** | **Sequence** | **Product Size**  **(bp)** | **Annealing Temp**  **(°C)** |
| --- | --- | --- | --- | --- |
| **AKT2** | Forward Outer | ACCTGAATCTCCAACCGC | 363 bp |  |
| **(RS2304 186)** |  |  |  |  |
|  | Reverse Outer | TAGTAGGGAGGTTTATTGAGATCG |  |  |
|  |  |  |  | **59°C** |
|  | Forward Inner (G Allele) | AAAAGGCTAAGTAAAAAG TTAGGAGG | 164 bp (Wild) |  |
|  | REVERSE inner (T Allele) | CCGCTGGGTGGTTTTCTA | 242 bp (mutant) |  |

**Table S3:** SNP rs2304186 correlation with demographic characteristics of blood cancer patients

| **Parameter** | **Genotype**  **/Allele** | **Categories** | | **OR (95% CI)** | **P-**  **value** |
| --- | --- | --- | --- | --- | --- |
| **Age** |  | **≤33**  **(n=157)** | **>33**  **(n=143)** |  |  |
|  | GG (W) | 63 (40) | 49 (34) | 1 | 1 |
|  | GT (H) | 72 (46) | 76 (53) | 0.7467 (0.4741 to 1.1762) | 0.2078 |
|  | TT (M) | 22 (14) | 18 (13) | 1.1317 (0.5798 to 2.2088) | 0.7169 |
|  | G | 198 (63) | 174 (60) | 1 | 1 |
|  | T | 116 (37) | 112 (40) | 0.9102 (0.6543 to 1.266) | 0.5761 |
| **Gender** |  | **Females**  **(n=136)** | **Males**  **(n=164)** |  |  |
|  | GG (W) | 53 (39) | 59 (36) | 1 | 1 |
|  | GT (H) | 64 (47) | 84 (51) | 0.8466 (0.537 to 1.3345) | 0.4732 |
|  | TT (M) | 19 (14) | 21 (13) | 1.1058 (0.5676 to 2.1545) | 0.7675 |
|  | G | 170 (63) | 202 (63) | 1 | 1 |
|  | T | 102 (37) | 126 (37) | 0.9619 (0.6906 to 1.3397) | 0.8183 |
| **Addiction** |  | **Smokers (n=159)** | **Non- Smokers (n=118)** |  |  |
|  | GG (W) | 59 (37) | 41 (35) | 1 | 1 |
|  | GT (H) | 83 (52) | 55 (47) | 1.2510 (0.7763 to 2.0159) | 0.3577 |
|  | TT (M) | 17 (11) | 22 (19) | 0.5224 (0.2636 to 1.0352) | 0.0627 |
|  | G | 201 (65) | 137 (58) | 1 | 1 |
|  | T | 117 (35) | 99 (42) | 0.8055 (0.5707 to 1.137) | 0.2187 |
| ***Leukemia*** |  | **Patients**  **(n=232)** | **Controls**  **(n=290)** |  |  |
|  | GG (W) | 85 (37) | 147(51) | 1 | 1 |
|  | GT (H) | 114 (49) | 119 (41) | 1.3883 (0.9804 to 1.9658) | 0.0645 |

|  | TT (M) | 33 (14) | 24 (8) | 1.8379 (1.053 to 3.2079) | 0.0322 |
| --- | --- | --- | --- | --- | --- |
|  | G | 284 (61) | 413 (71) | 1 | 1 |
|  | T | 180 (39) | 167 (29) | 1.5674 (1.2096 to 2.0312) | 0.0007 |
| ***Lymphoma*** |  | **Patients**  **(n=42)** | **Controls**  **(n=290)** |  |  |
|  | GG (W) | 16 (38) | 147(51) | 1 | 1 |
|  | GT (H) | 21 (50) | 119 (41) | 1.4370 (0.7513 to 2.7486) | 0.2732 |
|  | TT (M) | 5 (12) | 24 (8) | 1.1982 (0.3937 to 3.6466) | 0.7502 |
|  | G | 53 (63) | 413 (71) | 1 | 1 |
|  | T | 31 (37) | 167 (29) | 1.4465 (0.8967 to 2.3335) | 0.1303 |
| ***Myeloma*** |  | **Patients**  **(n=26)** | **Controls**  **(n=290)** |  |  |
|  | GG (W) | 11 (42) | 147 (51) | 1 | 1 |
|  | GT (H) | 13 (50) | 119 (41) | 1.4370 (0.6434 to 3.2096) | 0.3766 |
|  | TT (M) | 2 (8) | 24 (8) | 0.9236 (0.2057 to 4.1468) | 0.9174 |
|  | G | 35 (67) | 413 (71) | 1 | 1 |
|  | T | 17 (33) | 167 (29) | 1.2012 (0.6549 to 2.2034) | 0.5537 |
| **Marital Status** |  | **Married**  **(n=196)** | **Single**  **(n=104)** |  |  |
|  | GG (W) | 69 (35) | 43 (41) | 1 | 1 |
|  | GT (H) | 103 (53) | 45 (43) | 1.4521 (0.8998 to 2.3435) | 0.1266 |
|  | TT (M) | 24 (12) | 16 (16) | 0.7674 (0.3877 to 1.519) | 0.4473 |
|  | G | 241 (61) | 131 (63) | 1 | 1 |
|  | T | 151 (39) | 77 (37) | 1.0660 (0.7532 to 1.5085) | 0.7185 |
| **Residence** |  | **Urban**  **(n=110)** | **Rural**  **(n=190)** |  |  |
|  | GG (W) | 39 (35) | 73 (38) | 1 | 1 |
|  | GT (H) | 55 (50) | 93 (49) | 1.0430 (0.6521 to 1.6683) | 0.8605 |

|  | TT (M) | 16 (15) | 24 (13) | 1.1773 (0.5957 to 2.3269) | 0.6387 |
| --- | --- | --- | --- | --- | --- |
|  | G | 133 (60) | 239 (63) | 1 | 1 |
|  | T | 87 (40) | 141 (37) | 1.1088 (0.7883 to 1.5595) | 0.5530 |
| **Treatment** |  | **Treated**  **(n=225)** | **Untreated**  **(n=75)** |  |  |
|  | GG (W) | 88 (39) | 24 (32) | 1 | 1 |
|  | GT (H) | 106 (47) | 42 (56) | 0.6999 (0.4137 to 1.184) | 0.1834 |
|  | TT (M) | 31 (14) | 9 (12) | 1.1718 (0.5303 to 2.5896) | 0.6951 |
|  | G | 282 (63) | 90 (60) | 1 | 1 |
|  | T | 168 (37) | 60 (40) | 0.8936 (0.6121 to 1.3047) | 0.5602 |
| **Familial History** |  | **No History**  **(n=191)** | **Familial(n=109)** |  |  |
|  | GG (W) | 65 (34) | 47 (43) | 1 | 1 |
|  | GT (H) | 102 (53) | 46 (42) | 1.5696 (0.9764 to 2.5231) | 0.0627 |
|  | TT (M) | 24 (13) | 16 (15) | 0.8353 (0.4225 to 1.6514) | 0.6048 |
|  | G | 232 (61) | 140 (64) | 1 | 1 |
|  | T | 150 (39) | 78 (36) | 1.1605 (0.8221 to 1.6382) | 0.3975 |
| CI, confidence interval; OR, odds ratio; p-value ≤0.05 considered statistically significant; n, number of samples. | | | | | |


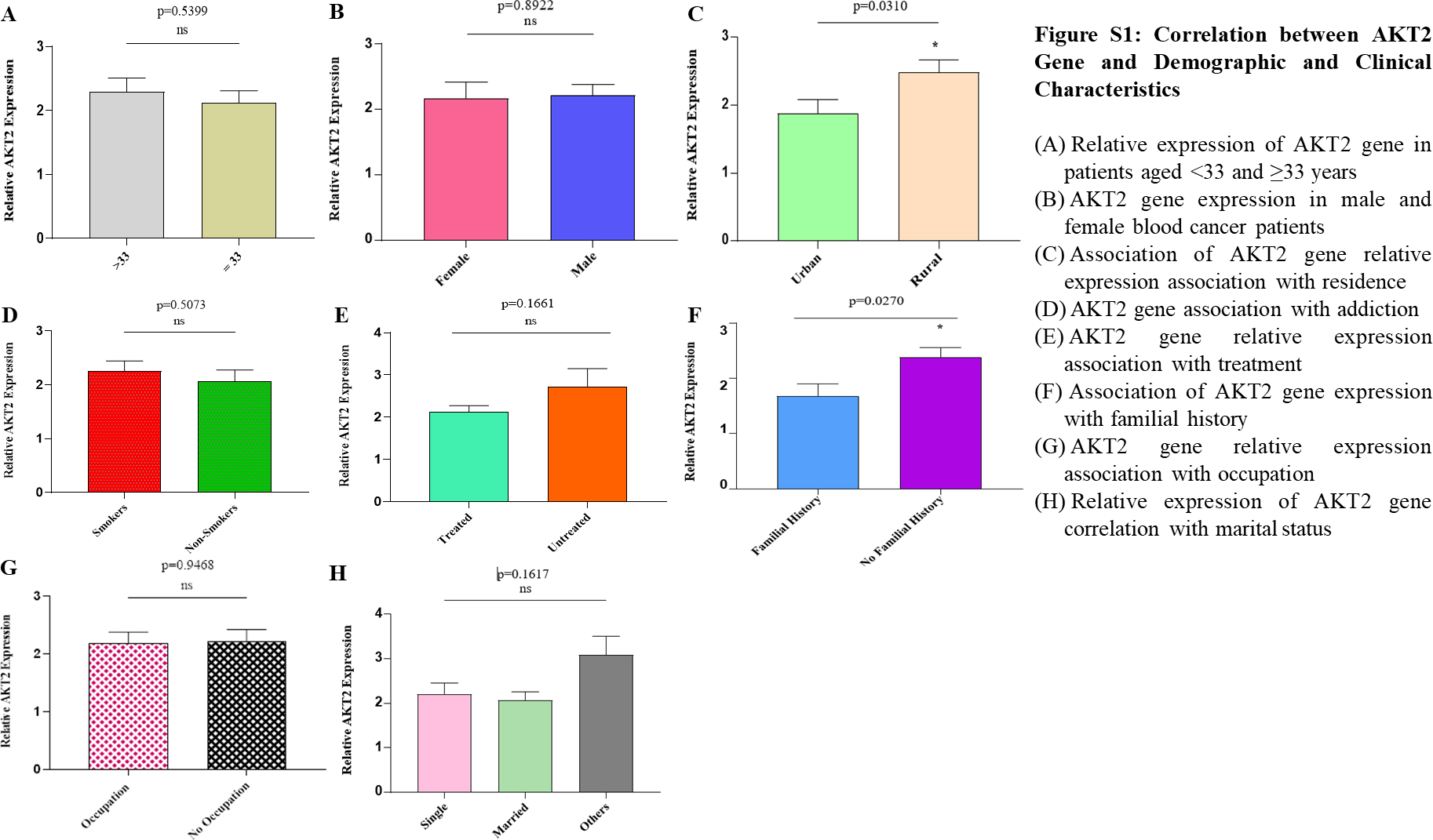


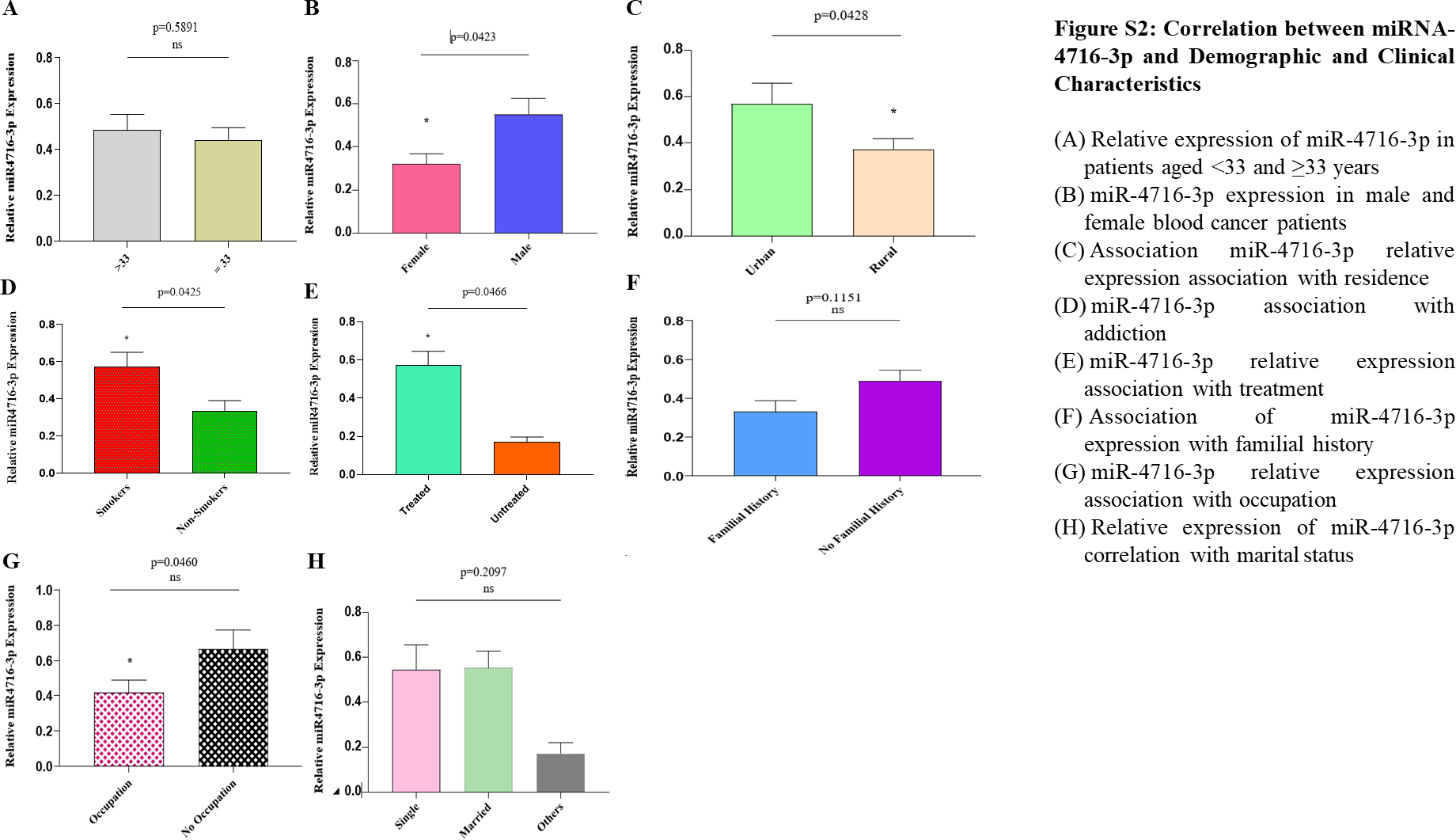


**A (Patients)**


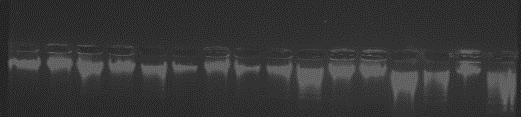


P17 P18 P19 P20 P21 P22 P23 P24 P25 P26 P27 P28 P29 P30 P31 P32

**B (Controls)**


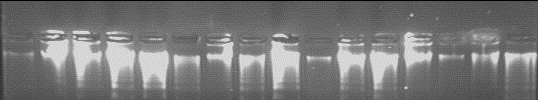


C81 C82 C83 C84 C85 C86 C87 C88 C89 C90 C91 C92 C93 C94 C95 C96

**Figure S3:** DNA samples electropherograms. (A) Blood cancer patients’ samples DNA,

(B) Control samples DNA.

**A (Blood Cancer Patients)**

**363 bp**


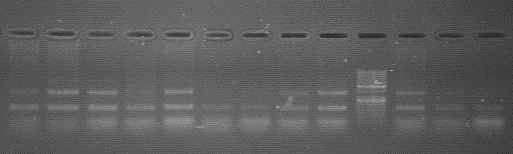


P35 P36 P40 P42 P43 P44 P51 P52 P60 L P62 P74 P76

**242 bp**

**164 bp**

**B (Healthy Controls)**

**363 bp 242 bp**


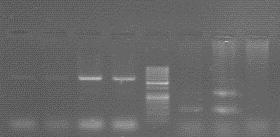


C1 C2 C3 C4 L C6 C7 C8

**164 bp**

**Figure S4:** Electropherograms of rs2304186 PCR-amplified products. (A) Polymorphism in blood cancer patients, (B) Control samples polymorphism.
